# Printing-Based Assay and Therapy of Antioxidants

**DOI:** 10.3390/antiox9111052

**Published:** 2020-10-28

**Authors:** Sera Hong, Baskaran Purushothaman, Joon Myong Song

**Affiliations:** College of Pharmacy, Seoul National University, Seoul 08826, Korea; happysr27@snu.ac.kr (S.H.); baskaran@snu.ac.kr (B.P.)

**Keywords:** antioxidants, 3D printing, inkjet printing, regenerative tissue

## Abstract

Antioxidants are essential in regulating various physiological functions and oxidative deterioration. Over the past decades, many researchers have paid attention to antioxidants and studied the screening of antioxidants from natural products and their utilization for treatments in diverse pathological conditions. Nowadays, as printing technology progresses, its influence in the field of biomedicine is growing significantly. The printing technology has many advantages. Especially, the capability of designing sophisticated platforms is useful to detect antioxidants in various samples. The high flexibility of 3D printing technology is advantageous to create geometries for customized patient treatment. Recently, there has been increasing use of antioxidant materials for this purpose. This review provides a comprehensive overview of recent advances in printing technology-based assays to detect antioxidants and 3D printing-based antioxidant therapy in the field of tissue engineering. This review is divided into two sections. The first section highlights colorimetric assays using the inkjet-printing methods and electrochemical assays using screen-printing techniques for the determination of antioxidants. Alternative screen-printing techniques, such as xurography, roller-pen writing, stamp contact printing, and laser-scribing, are described. The second section summarizes the recent literature that reports antioxidant-based therapy using 3D printing in skin therapeutics, tissue mimetic 3D cultures, and bone tissue engineering.

## 1. Introduction

Antioxidants are substances that can inhibit free radical-mediated oxidative stress and toxic side effects in the human body. The free radicals (e.g., reactive oxygen species and reactive nitrogen species) are formed in vivo as a result of normal metabolic processes or external exposure like X-rays, UV, ozone, air pollutants, cigarette smoke, etc. [1,2]. The free radicals are highly unstable and reactive, so they can damage various endogenous systems by attacking important biological molecules including lipids, nucleic acids, and proteins [3]. The human body has endogenous antioxidant defenses (enzymatic like glutathione peroxidase (GPx), catalase, or non-enzymatic like bilirubin, uric acid) that remove reactive species; however, it is insufficient to prevent damage completely [4]. If the body antioxidant defense system fails to neutralize the excess free radicals, the imbalance between oxidants and the defense system can lead to pathological conditions, including cancer [5], heart disease [6,7], neurodegenerative disorders [8,9], atherosclerosis [10], diabetes [10], and asthma [11]. Thus, exogenous antioxidants are significant for sustaining health. The effects and actions of antioxidants in vivo have been extensively investigated in multidisciplinary areas [12,13] but are not be covered in detail here.

Antioxidants are mostly obtained from plants and plant-derived compounds (e.g., polyphenols, flavonoids, carotenoids, and vitamin E, A, C). Reliable screening and evaluation of antioxidants in various plants have been a central issue for their useful applications. Various analytical methods, including chromatography, spectrophotometry, capillary electrophoresis, and electrochemical sensors have been conventionally used to this end [14,15]. Amongst them, screen-printed electrodes (SPEs)-based electrochemical sensors are one of the attractive techniques to measure antioxidants and related properties due to their simplicity, low cost, rapidity, small size, and portableness. The screen-printed electrodes are manufactured via screen printing technology by depositing conductive inks layer-by-layer upon a solid substrate [16]. The main advantage associated with SPE based sensors is the easy modification of the surface of the electrode to identify various antioxidants in different types of samples (e.g., wine, meat, oil, tablet, skin, etc.) [17,18,19,20,21,22]. There are wide varieties of modifiers, and modifying the SPEs with various modifiers enhances their sensing capability by providing special properties to SPEs for diverse applications. The modifiers mainly used for detecting antioxidants are enzyme, polymer, and nanomaterials (e.g., carbon nanotubes (CNTs), graphene-based inks, metal, or metal oxide nanoparticles, carbon black (CB), transition metal dichalcogenides (TMDs), etc.) [23,24,25,26,27]. The modification of SPEs can be easily implemented by altering the printing ink composition with different substances or depositing various materials on the surface of the working electrodes [16]. Circumspect choice of the modifiers can significantly improve the overall electrode performance by offering more excellent reproducibility, stability, sensitivity, and selectivity. Especially, a notable advancement in the development of SPEs for the detection of antioxidants has achieved using different nanomaterials. Carbon nanomaterials such as CNTs and graphene-related materials are very attractive electrode modifiers as they accelerate the electron transfer rate on the electrode surface and increase the electrode’s surface area, reducing the passivation of the electrode area [23,28]. Nanomaterials like metal nanoparticles and CNTs are a suitable candidate for bio-functionalization since they are compatible and readily bind to a variety of biomolecules [25]. In recent years, the use of new nanomaterials, such as carbon black (CB), Printex L6 carbon (PC), and transition metal dichalcogenides (TMDs), has been demonstrated for the advanced SPEs modification for antioxidants detection [24,26,27]. CB and PC are a low-cost, nanostructured alternative to CNTs and graphene. They have notable features of fast electron transfer and high electroactive surface area, comparable to CNTs and graphene. On the other hand, transition metal dichalcogenides (TMDs) have attracted increasing interest as a modifier for hydrogen evolution reaction (HER) and capacitive energy storage [29,30]. In particular, the use of layered transition-metal dichalcogenides, i.e., WS_2_ and MoS_2_, is a growing field for constructing electrode for antioxidant detection by reducing the passivation of the electrode area [24]. 

Apart from the screen-printing technology, the application of inkjet-printing technology has also been reported for an antioxidant colorimetric assay [31,32]. Inkjet printing has been widely utilized to directly deposit various substrates like DNA, protein, and nanomaterials [33]. In particular, the noncontact deposition of inkjet printing is appropriate for printing biomaterials by maintaining the native conformation of biomaterials during the printing process, compared to other deposition methods such as vapor deposition or bioMEMS [31,34,35]. In this regard, recent studies have successfully shown that the antioxidant activities of test substances could be evaluated through the inkjet-printed enzyme inhibition assays based on colorimetric detection [31,32]. The inkjet printing has excellent properties such as the consumption volume of reagents from nanoliters to picoliters, accurate placement of ejected materials, and high-throughput process. These properties not only reduce the time and cost required to prepare a sufficient amount of analytes from natural products but also enable automatic screening assays without labor-intensive steps, compared to the traditional well plate method [31,32,34]. Although the print head is prone to clogging and ink bleeding, the inkjet printing-based approach is expected to provide significant development in investigating antioxidants in various ways.

Until comparatively lately, many conventional studies have focused on elucidating antioxidants and their capacity from various natural products and introducing them in health supplements or food industries. However, as three-dimensional (3D) printing technology has evolved over the past decade, the application of antioxidants has recently expanded into biomedical engineering for antioxidant therapy. 3D printing technology is the process of creating 3D objects from a digital design file by adding material layer-by-layer. The major advantages of 3D printing technology include rapid prototyping, cost-effectiveness, flexibility, high quality, and consistency [36,37]. These advantages of 3D printing used for treatments with antioxidants can show a synergistic effect in medicine [38,39,40,41,42,43]. The applications of 3D printing in the field of medicine are various including device fabrication, implants, scaffolds for tissue engineering, 3D cancer models, and drug delivery systems [43,44]. Recent studies have demonstrated that 3D printed scaffolds conjoined with antioxidant substances provide good characteristics like radical scavenging, anti-cancer, and anti-inflammatory activities [38,40,41]. Furthermore, the antioxidants containing 3D scaffolds significantly influence the cell viability and successful tissue growth in the 3D printed scaffolds [38,41]. 

This review consists of two main sections. The first section is devoted to summarizing the development of printing-based antioxidant assays, both colorimetric and SPE based electrochemical assays. The second section discusses the advances of 3D printing-based antioxidant therapy and is organized into the sub-categories of skin therapeutics, tissue mimetic 3D cultures, and bone tissue engineering.

## 2. Printing-Based Antioxidant Assays

### 2.1. Colorimetric Assays

Due to the importance of antioxidants in the protection of free radicals which may cause food spoilage or many disorders such as heart disease and cancers in humans, antioxidants have been widely investigated as a potential drug candidate or preservative using a variety of testing methods. The testing methods generally include the 2,2-diphenyl-1-picrylhydrazyl (DPPH) method, 2,2′-azino-bis(3-ethylbenzothiazoline-6-sulfonic acid) (ABTS) assay, ferric reducing/antioxidant power (FRAP) assay, etc. based on free radical scavenging [45]. These antioxidant assays are based on the inhibition of the absorbance of the radicals by antioxidants. When the stable absorbance (e.g., DPPH^•^ at 520 nm, ABTS^•+^ at 415 nm, etc.) is obtained, the antioxidants sample is added to the radical solution, and then the scavenging ability of antioxidants is measured in terms of decolorization [45]. These antioxidant assays are relatively simple and widely used for different antioxidant studies. However, in the process of determining the antiradical scavenging activity of antioxidants, a considerable amount of analytes and reagents is consumed. Moreover, laborious effort and considerable expense are required to obtain a sufficient number of samples due to the yield limit of production by synthesis or isolation from natural products [46]. Inkjet printing is a contact-less, rapid, and cost-effective printing technology [47]. Recently, an inkjet printing-based colorimetric assay has been reported in the antioxidant screening process by depositing biomolecules on paper-based substrates [31,32]. The inkjet printing-based assay could be a suitable method to overcome the problem of the consumption of unnecessary reagents thanks to its advantage of a very small ejection volume, i.e., from nanoliters to picoliters. In addition, a contactless and heat-free deposition system of inkjet printing enables the handling of bioactive or heat-sensitive materials like enzymes or some vitamins in a highly reliable manner.

**Figure 1:**
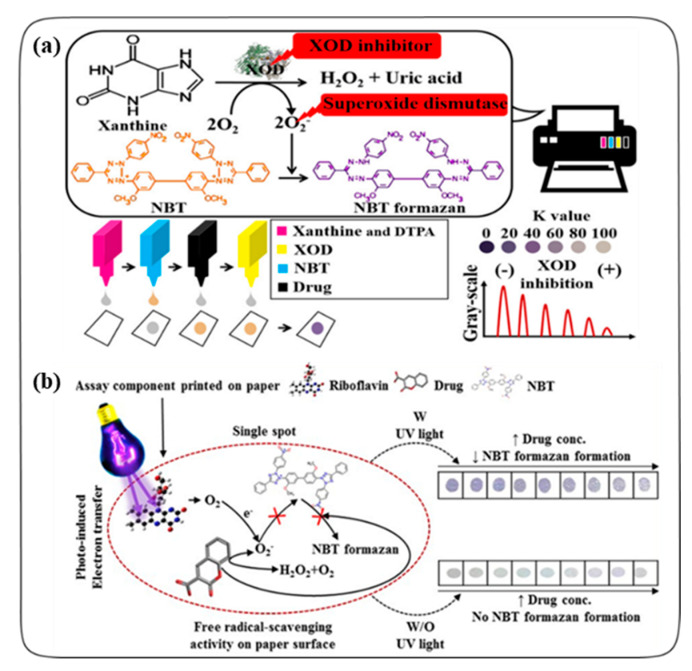
(**a**) Schematic representation of inkjet printing-based xanthine oxidase (XOD) inhibitory assay. Reprinted with permission from Ref. [31]. Copyright (2017) American Chemical Society. (**b**) Schematic illustration of inkjet printing-based radical scavenging assay on paper. Reprinted with permission from Ref. [32]. Copyright (2018) Elsevier.

Song et al. described an inkjet-printed enzyme inhibitory assay for screening potential antioxidant agents using a commercially available HP inkjet printer, as shown in Figure 1a [31]. Their method determined the inhibitory mole (IM50) value of the test compounds to quantitatively evaluate the inhibitory activity of a xanthine oxidase (XOD) through the colorimetric estimation of nitro blue tetrazolium (NBT) formazan. Four printer cartridges were filled with ink solutions of xanthine, NBT, drug (i.e., aminoglutethimide, dithranol, naringenin, or superoxide dismutase as a control), and xanthine oxidase, respectively, and continuously ejected onto A4 paper (the reaction spot was 0.3 cm in diameter). The amount of drug to be printed could be controlled by adjusting the K value in the printer setup as a parameter ejection volume using Adobe Photoshop software. Thereby, the XOD inhibitory activity of the test drug was evaluated in a dose-dependent manner. Drugs with an antioxidant capacity reduced the amount of superoxide anions (O^2−)^ that act as an NBT reducer, resulting in decreased absorbance of NBT formazan (purple) at 560 nm scanned with an HP scanner. The consumed amounts of the reaction components in the inkjet-printed molecular assay were 2–3 orders of magnitude smaller than those of the well-plate-based absorption assay, and the reaction time of the inkjet printing-based method was more rapid compared to the conventional well-plate-based method. 

In addition to these findings, Song et al. reported an inkjet-printed radical scavenging assay using the photo-induced electron transfer (PET) reaction to investigate the radical scavenging potential of natural plant products (Figure 1b) [32]. In that study, the photo-induced electron transfer (PET) process involved the use of riboflavin for PET and an ultraviolet lamp (362 nm) as a light source. Superoxide anions (O^2−^) generated from the UV-irradiated riboflavin converted NBT (yellow) to NBT formazan (purple). Riboflavin, NBT, and the natural compounds (i.e., scopoletin, umbelliferone, aesculetin, or coumarin) were serially deposited on the parchment paper with a conventional inkjet printer. Parchment paper was used for assisting the interaction of the components without self-absorption. Natural compounds with a radical scavenging activity inhibited NBT reduction in proportion to the concentration of the compound in the presence of UV irradiation. That study indicated that the inkjet printing-combined molecular assay minimized manual and time-consuming tasks (such as repeated pipetting) and consumed less sample volume 4- to 5-orders of magnitude compared to the well-plate-method. Thus, the inkjet-printed radical scavenging assay enabled a comfortable and cost-effective molecular assay approach based on a photochemical reaction.

### 2.2. Electrochemical Assays 

Screen-printed electrode (SPE)-based electrochemical sensors perform rapid and sensitive detection of interesting analytes. Many previous studies have shown that various electrochemical methods have been successfully applied to determine antioxidant activities in different kinds of samples [48]. SPEs usually consist of a three-electrode system (working, counter, and reference electrode) where various materials such as ceramic, plastic, paper, or glass are printed [16]. Surface modification of electrodes with an extensive range of substances (e.g., metals, enzymes, polymers, metal nanoparticles, carbon nanotubes, graphene-based inks, etc.) is the most widely used strategy to enhance selectivity, sensitivity, and reactivity required for the detection of antioxidants [23,49,50]. A recent study has demonstrated that electrodes coated with multiwalled carbon nanotubes (MWCNT) embedded within a conducting polymer poly(3,4-ethylenedioxythiophene) (PEDOT) possess greater sensitivity, a lower limit of detection (LOD), and excellent stability over a period of 2 months, compared to standard glassy carbon electrodes for the determination of antioxidants in beverages [51]. In this section, electrochemical detections of antioxidants from various samples (e.g., foods, tablets, and skin) using the SPEs and their properties are introduced based on the papers published from 2015 to 2020. The various electrochemical detections have been attempted using the modifications of SPEs: (i) nanomaterials; carbon nanotubes, metal or metal oxide nanoparticles, and cutting-edge nanomaterials (carbon black, Printex L6 carbon, transition metal dichalcogenides), (ii) enzymes, (iii) polymers. The characteristics and distinction of each method are introduced in this section. (iv) Alternative screen-printing techniques, such as xurography, roller-pen writing, stamp contact printing, and laser scribing, are described. As a result of the page limitation, some major papers in this area may not be included in this chapter. 

#### 2.2.1. Nanomaterials

##### Carbon Nanotubes

Carbon nanotubes (CNTs) are an efficient chemical modifier of the SPE surface. Carbon nanotubes have attractive properties including a high electroactive area and mechanical strength [52]. The shape of the carbon nanotubes with multiple corners and sharp edges also provides excellent electrocatalytic activity [53,54]. For these reasons, carbon nanotube-modified SPEs have been extensively used for the development of electrochemical sensors to increase the electron transfer properties and surface area of the electrode [55]. Carbon nanotubes exist in two main forms: single-walled carbon nanotubes (SWCNTs) and multi-walled carbon nanotubes (MWCNTs). A single-walled carbon nanotube has a hollow cylindrical structure consisting of a single layer of allotropes of carbon, while a multi-walled carbon nanotube is comprised of several SWCNTs [56]. The distinction between SWCNTs and MWCNTs relies on the difference in diameter and flexibility [56]. MWCNTs are more rigid and larger than the SWCNTs. In recent years, many efforts have been given toward developing high-performance sensors for antioxidant sensing using CNT-modified SPEs.

**Figure 2:**
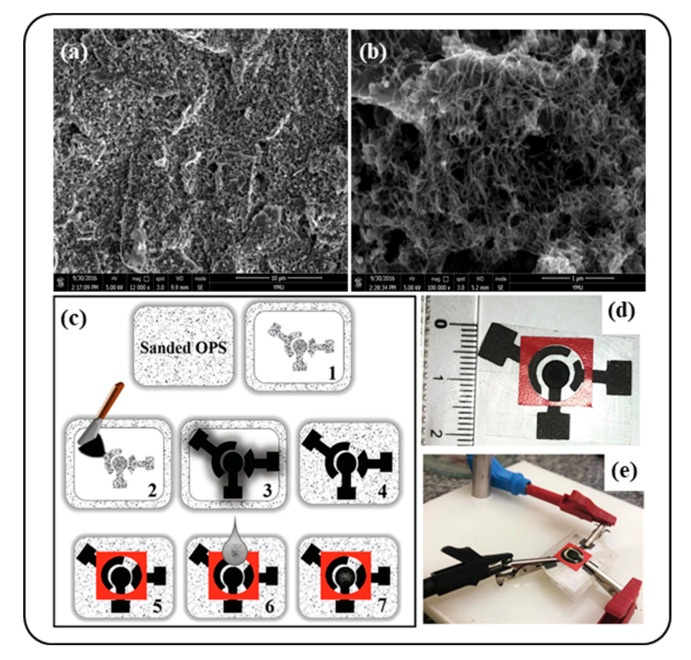
(**a**) The SEM characterization of the bare screen-printed electrode (SPE) and (**b**) the multi-walled carbon nanotubes modified screen-printed electrode. Reprinted from Ref. [57] with permission from MDPI. Schematic representation of (**c**) SPE preparation, (**d**) images of the final device, and (**e**) the final SPE connected to the potentiostat. Reprinted with permission from Ref. [59]. Copyright (2020) Elsevier.

Gao et al. fabricated multi-walled carbon nanotubes modified screen-printed electrode (MWCNTs/SPE), which detected the antioxidant substance chlorogenic acids (CGAs) [57]. Chlorogenic acids are a polyphenolic compound common in plant materials, especially in coffee. This compound is known as an antioxidant that could protect against cancer, diabetes, liver disease, and neurodegenerative disorders like Parkinson’s disease. The MWCNTs/SPEs were prepared by coating the MWCNTs suspension onto commercial SPEs, and the differential pulse voltammetry (DPV) method was used to determine the CGAs. The MWCNTs distributed uniformly on the surface of the SPE formed a structure of entangled cross-linked fibrils, which could offer a greatly expanded surface area of the electrode. Figure 2a,b show the SEM image of the MWCNTs modified SPEs compared to the bare SPE. Under the optimal conditions, the linear ranges were from 0.17 to 16 μg/mL and the detection limit for CGAs was 0.12 μg/mL. This proposed method was quick and highly sensitive and can be applied to the estimation of CGA in the real samples (e.g., coffee beans). 

Furthermore, the polyphenol content and antioxidant capacity of red wines were satisfactorily analyzed by using a single-walled carbon nanotubes-modified screen-printed carbon electrode (SWCNTs-SPCE) with square wave voltammetry (SWV) [58]. When three different types of electrodes, namely glassy carbon electrode (GCE), SWCNT, and MWCNT were compared as a working electrode, the best results were obtained especially in the SWCNT electrode. The SWCNT promoted electron transfer for the electrochemical reactions. The total polyphenols content determined using SWCNT-SPCE were well-matched with the value obtained by worldwide analytical techniques such as the ABTS method and spectrophotometric and chromatographic methods. Therefore, the SWCNTs-SPCE based analysis with the application of SWV can be a quick and easy method for polyphenol characterization and antioxidant capacity determination.

Santos et al. demonstrated a lab-made screen-printed electrode modified with functionalized multi-walled carbon nanotubes (MWCNTs/SPEs) on the quantification of caffeic acid in tea samples [59]. Carboxylic acid groups-functionalized MWCNTs were used in this platform. As shown in Figure 2c–e, for the preparation of disposable SPEs, first, a conductive ink containing nail polish and graphite was deposited on polyester overhead projector sheets (OPS). Then, a carbon nanotube-modified working electrode was made by a simple drop-casting method using a zein/MWCNTs suspension. The results of the developed SPE by voltammetry measurement showed a reliable determination of CA in the tea samples with a broad linear range (2.0–50 μmol/L) and low limits of detection (0.2 μmol/L and quantification (0.66 μmol/L). Moreover, one of the advantages of this technique is that the electroanalysis involved minimal consumption of the sample and chemicals (only 100 μL of solution).

##### Metal or Metal Oxide Nanoparticles

The use of metal or metal oxide NPs for SPEs can be frequently observed in many previous studies [21,22,60]. Applying metal or metal oxide nanoparticles (NPs) to SPEs brings about many advantages. Some metallic NPs (e.g., platinum, rhodium, iridium, etc.) have electro-catalytical properties towards analytes by acting as transducers for enzymatic reactions or as a non-enzymatic sensor [61]. Other metallic NPs can also function as anchorage substrates. These unique electronic and catalytic properties of metallic nanoparticles are derived from their small size and large surface-to-volume ratio [62]. Especially, gold NPs are a suitable candidate for bioconjugation, because they can be easily functionalized and also changed in size, shape, and nature [63,64]. Metal and metal oxide nanoparticles can be used in combination with various other conductive materials (e.g., carbon nanomaterials) to achieve synergistic physical and chemical properties like heterogeneous electron transfer rate and increased electrical contact surface [62]. Among the industrially utilized materials, graphene has gained much attention as a good support platform for them. Focusing on the field of sensing antioxidants in various samples, only a few works using metal or metal oxide NPs-based SPEs are discussed here. 

M.A. Ali et al. reported a voltammetric sensor based on a screen-printed carbon electrode (SPCE) altered using reduced graphene sheets decorated with cobalt diselenide nanoparticles (CoSe2@rGO) to detect a propyl gallate in meat [21]. The propyl gallate is a phenolic antioxidant that is widely used as a food preservative to protect oils and fats from oxidation. However, recent studies have shown that the use of PG is harmful to health because it is related to carcinogenesis and liver damage. In that work, a stable and high-performance electrode was manufactured using the CoSe2@rGO nanocomposite for rapid, sensitive electrochemical sensing of PG in real samples. The cobalt diselenide (CoSe2) possesses a good conductivity as an electrocatalyst, and the reduced graphene oxide (RGO) can enhance the electrochemical performance of the CoSe2. The working electrode was made by drop-casting the CoSe2 and rGO dispersion on SPCE. At an optimal state, the modified SPCE exhibited an excellent capability with a low detection limit of 16 nM and high sensitivity. Therefore, this CoSe2@rGO electrode-based sensor can be used to detect PG for food safety. 

**Figure 3:**
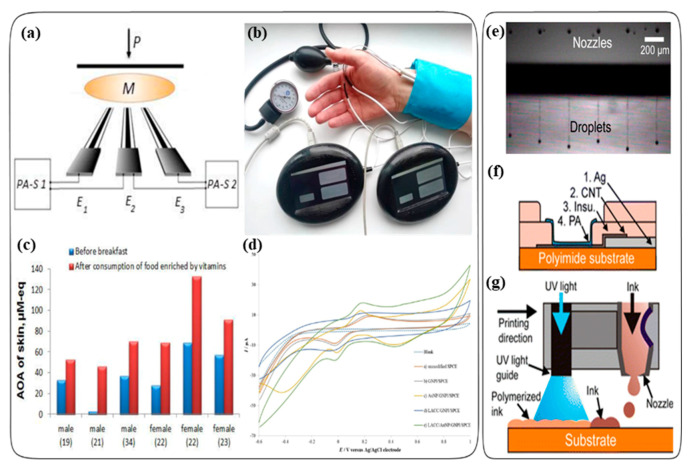
(**a**) Measuring scheme. (**b**) Photo illustrating the monitoring process of skin antioxidant activity (AOA) using a sensory system. E1–E3: electrodes; M: membrane; PA-S 1 and PA-S 2: potentiometric analyzers; P: load. (**c**) Measurement of the enhancing effect of consumption of food enriched with vitamins on the skin AOA of 6 volunteers (The age of the respondents is indicated between parentheses). Reprinted from Ref. [22] with permission from MDPI. (**d**) Cyclic voltammograms of hydroquinone on different modified electrodes; blank with an unmodified SPCE (a), GNPl/SPCE (b), AuNP/GNPl/SPCE (c), LACC/GNPl/SPCE (d), LACC/AuNP/GNPl/SPCE (e). Reprinted with permission from Ref. [49]. Copyright (2019) Elsevier. (**e**) Stable droplet formation obtained using six nozzles simultaneously, (**f**) Schematic illustration of inkjet-printed PA/CNT electrodes based on successive printing of Ag, CNT, insulator, and PA, (**g**) Schematic representation of simultaneous IJP and UV photopolymerization. Reprinted with permission from Ref. [18]. Copyright (2015) American Chemical Society.

Brainina et al. developed a new disposable sensor system for monitoring the oxidative stress of skin using an antioxidant activity (Figure 3a,b) [22]. Oxidative stress of skin is developed by internal and external factors of the skin, such as the production of reactive oxygen species (ROS), and exposure to ultraviolet (UV) radiation. This leads to damage of the skin’s structural elements, skin aging, and skin cancer. These harmful effects should be balanced or controlled by the antioxidant system of the skin. Thus, monitoring the antioxidant activity of the skin is an important task to diagnose skin related diseases. In that study, they developed a carbon or silver printed electrode as the sensing device for monitoring the skin antioxidant activity. Briefly, they fabricated the modified carbon-screen printed electrode (CSPE) and silver screen-printed electrode (AgSPE) with a manual screen printing device. Glass fiber was used as a substrate for the screen-printed electrodes. The working area of the electrode was created with a mixture of Cementit and acetone, and then, a gold nanoparticle (AuNP) suspension was applied onto that. After drying, the AuNP coated CSPE indicator electrode for the sensory system was prepared. The sensory system was used with a small group of people to evaluate the presence of oxidative stress in the skin. They evaluated the impact of fasting and the consumption of food and food enriched by vitamins (antioxidants) on the skin antioxidant activity. The antioxidant activity was evaluated before and after breakfast. They observed enhanced antioxidant activity in the skin of 6 volunteers after the consumption of food enriched by vitamins (Figure 3c). The observed increase in the skin antioxidant activity after meals enriched with vitamins (vitamin C 50 mg and rutoside 50 mg), showed that there is a relationship between the internal environment of the body and the skin surface. The developed device in this study can be used for on-site and in situ measurements of antioxidant activity. 

Puangjan and Chaiyasith devised an efficient ZrO_2_/Co_3_O_4_/reduced graphene oxide nanocomposite electrochemical sensor for the simultaneous determination of natural antioxidants in fruit juice, rice, and tea samples [65]. The idea of this report is combining metal-oxide nanoparticles (ZrO_2_, Co_3_O_4_) with graphene to fabricate a modified electrode with good electrocatalytic activity. Zirconium dioxide (ZrO_2_) nanoparticles have good catalytic activity, chemical inertness, thermal stability, and lack of toxicity. Cobalt oxide (Co_3_O_4_) nanoparticles also have been applied to the development of various sensors due to its good catalytic efficiency. The ZrO_2_/Co_3_O_4_/reduced graphene oxide (rGO) nanocomposite catalyst was prepared by the reflux method, then the nanocomposite was cast onto the surface of the fluorine-doped tin oxide (FTO) electrode. This proposed electrode showed a satisfactory catalytic effect toward oxidation of gallic acid (GA), caffeic acid (CA), and protocatechuic acid (PA) with linear ranges of 6.0 × 10^0^–4.8 × 10^2^ nmol L^−1^, 2.0 × 10^0^–5.2 × 10^2^ nmol L^−1^, 5.0 × 10^0^–4.2 × 10^2^ nmol L^−1^, and limits of detection of 1.6 nmol L^−1^, 0.62 nmol L^−1^, and 1.4 nmol L^−1^ for GA, CA, and PA.

##### Cutting-Edge Nanomaterials

Carbon black (CB) is a low-cost nanostructured material made from petroleum products combustion. In recent years, CB became an interesting modifier for building electrochemical platforms due to its high surface area, high electrical conductivity, low cost, and high dispersibility [66]. Arduini et al. reported a miniaturized and disposable sensor for phenolic compound detection by using CB-modified SPEs [27]. The CB-SPE exhibited higher sensitivity without fouling problem than the bare SPE for phenolic compound detection by square wave voltammetry. Catechol, gallic acid, caffeic acid, and tyrosol were detected with a LOD of 0.1 μM, 1.0 μM, 0.8 μM, and 2.0 μM, respectively, and linear range of 1.0–50 μM, 1.0 × 10^1^–1.0 × 10^2^ μM, 1.0–50 μM, and 1.0 × 10^1^–1.0 × 10^2^ μM, respectively. Furthermore, CB-SPE could be used for distinguishing between mono-phenol (tyrosol) and ortho-diphenols (caffeic acid) in the voltammograms. The developed sensor confirmed the high potentiality of CB as an attractive alternative modifier compared to the most used graphene and carbon nanotubes.

On the other hand, Printex L6 nano-carbon (PC) is another promising candidate as a modifier appropriate for carbon nanotubes and graphene, which has better heterogeneous electron transfer constant, improved sensitivity, and low background current. Raymundo-Pereira et al. synthesized a Printex nano-carbon and silver hybrid nanomaterial (PC-Ag) for the estimation of antioxidant activity [26]. The hybrid nanomaterial (PC-Ag) was prepared directly by applying the silver nanoparticles (AgNPs) on the surface of the PC using ethylene glycol as the reducing agent. At an optimal condition, the PC-AgNP-modified glassy carbon electrode represented good efficiency and sensitivity for the estimation of total polyphenols in wine. The synergetic effect between PC nanocarbons and AgNP led to oxidation of gallic acid (GA) at lower potentials, yielding higher current responses. In cyclic voltammetry, GA was confirmed with a linear range of 0.5–8.5 nmol L^−1^ and LOD of 66 nmol L^−1^

Transition metal dichalcogenides (TMDs) are a family of layered compounds with the formula MX_2_, where M is a transition metal atom (Mo, W, etc.) and X is a chalcogen atom (S, Se, Te, etc.). TMDs hold great promise in sensor development for antioxidant detection. In particular, TMDs-based nanocomposites have attracted researchers’ attention due to their excellent anti-fouling properties towards polyphenols [30]. Electrode fouling is a serious problem encountered in some electrochemical analyses since fouling prevents an analyte of interest from making physical contact with the electrode [67]. An impermeable layer formed on the electrode by fouling leads to an electrode surface’s passivation and thereby reduces sensitivity and reproducibility [68]. Recently, Escarpa and Compagnone group fabricated an electrochemical sensor based on the synergic employment of MoS_2_ and CB for polyphenolic catechins determination in cocoa [24,68]. They merged CB ability to increase electrochemical sensitivity and the MoS_2_ antifouling effects against catechins. The SPE-CB/MoS_2_ showed an impressively stable and reproducible response and excellent resistance to the fouling problem that occurs to conventional carbons and nanomaterials-modified electrodes due to the catechins by-products of strong oxidation tendency to interact with the electrode. Moreover, they devised a DMSO-based solubilization extraction-free strategy for fast (required only 15 min), direct, and eco-friendly polyphenols content evaluation. The results showed that SPE-CB/MoS_2_ improved the sensitivity (LOD ≤ 0.17 μmol L^−1^) of 100-folds compared to the bare SPE electrode, showing a linear range between 0.12 and 25 μmol L^−1^.

#### 2.2.2. Enzymes

Enzyme-modified electrodes have been developed intensively in the last few decades for the construction of electrochemical biosensors. The immobilization of enzymes on screen-printed sensors via various techniques (such as casting, physical adsorption, or electrochemical coating) has been used for the estimation of antioxidant analytes or total antioxidant capacity (TAC) in various kinds of biological samples like foods [49,69], plant extracts [70], plasma [71], pharmaceutical compounds [72], etc. The sensitivity of electrochemical biosensing could be amplified through redox enzyme modification of SPEs because antioxidants interacting with an active enzyme redox center generate better current responses than unmodified electrodes [49,73]. The immobilized redox enzymes facilitate biological electron transfer processes and confer specificity and selectivity to the devices as a recognition element [74]. In this regard, a wide range of enzymes for reducing antioxidant substrates (such as laccase, tyrosinase, cytochrome c, etc.) has been widely used to design electrochemical biosensors with high selectivity, activity, and stability [74]. Although many types of enzymatic biosensors based on different modified SPEs and immobilization methods have been demonstrated for the determination of antioxidants, only representative examples are briefly introduced here. 

Csiffary et al. devised an amperometric biosensor for the determination of L-ascorbic acid (vitamin C) in different fruit juices and vitamin C effervescent tablets using an ascorbate oxidase enzyme (AAO)-modified screen-printed carbon electrode [19]. The AAO enzyme was immobilized on the electrode with a cross-linker poly (ethylene glycol) (400) diglycidyl ether (PEGDGE). The amperometric response of the sensor was measured and compared with three different electrodes (blank electrode, inert protein-bovine serum albumin (BSA) electrode, and AAO electrode). The enzymatic degradation of ascorbic acid by ascorbate oxidase caused a decreased electrochemical signal in the sensor compared to the measurement without ascorbate oxidase. The detection limit of L-ascorbic acid was 3μmol/L, and the linear measuring range was from 5.0 × 10 to 1.5 × 10^2^ μmol/L. This sensor had a high sample frequency (30 samples per hour), good reusability (up to 200 samples), and easy sample preparation compared to the several AAO-based sensors developed earlier in the literature. 

Besides simply applying enzymes to the SPE surface, there recently have been increasing reports of biosensors whose conductivity and stability were enhanced by enzyme-modified SPEs together with gold nanoparticles. For example, Bernalte et al. explored an application of tyrosinase-modified gold nanoparticles screen-printed electrodes (Tyr-AuNPS-SPCEs) for direct amperometric analysis of polyphenols in commercial beers [69]. Tyrosinase is a monophenol oxidase that mainly is involved in the oxidation of phenol via catechol to o-quinone. Extensive studies have been conducted on electrochemical detection of phenolic compounds using the tyrosinase enzyme. In that study, the proposed methodology showed good analytical and kinetic performance for the estimation of antioxidant phenolic compounds (i.e., catechol, phenol, caffeic acid, and tyrosol) in complex beer samples. Because gold nanoparticles (AuNPs) possess excellent electrical properties and large active surface areas, the authors utilized gold nanoparticles not only as robust support for enzyme deposition on a commercial screen-printed electrode but also as an electrical conductance enhancer, thereby improving the sensitivity of the biosensor. The Tyr-AuNPS-SPCEs were fabricated by sequential drop-casting of the tyrosinase enzyme solution to immobilize the enzyme on the AuNPS-SPCEs surface, followed by the addition of glutaraldehyde solution for crosslinking. The analyzed results of the phenolic content with the developed biosensor represented a good Pearson correlation (r = 0.821, *p* < 0.01, 99%) when compared with the results obtained by a standardized Folin–Ciocalteau spectrophotometric method. 

Pavinatto et al. designed an inkjet-printed interdigitated electrode (IDE) as a sensor for the detection of antioxidants in wines and olive oils [20]. They fabricated gold (Au) interdigitated electrodes (IDEs) as a biosensor, which were directly inkjet-printed onto substrates using an Au nanoparticle-based ink. The Au ink NPG-J was directly printed on specially coated plastic substrates such as polyethylene naphthalate (PEN, PQA1). The Au IDE electrode was used for antioxidant measurements, which showed higher oxidation potential and stability in a biological medium. Once the Au IDEs were made, the tyrosinase (Tyr) enzyme was used in the active layer of the electrodes. The tyrosinase ink was formulated using the enzyme stabilizer trehalose, triton x-100 as a non-ionic polymeric surfactant, and carboxymethyl cellulose as a viscosity enhancer. The Tyr ink was gravure-printed on a plastic substrate (PQA1) which contains the Au ink-jetted electrode (Au IDE), providing homogenous patterned films. Then finally the Tyr ink coated Au IDE electrode was tested using pyrogallol (Pyr) as a model antioxidant with an electrical impedance detection method. Tyr catalyzed Pyr to purpurogallin (Pur) and then formed a dianion of purpurogalloquinone via peroxide oxidation. The data showed that the electrical impedance signal increased linearly with the Pyr concentration. This increased electrical impedance confirmed that the printed biosensor could efficiently detect antioxidants in solutions. Furthermore, they also did impedance measurements with the Au IDE electrode with and without the Tyr enzyme deposited on the Au IDE electrode. The data showed that the impedance signal of the Tyr enzyme printed Au IDE increased according to the Pyrogallol concentration. However, the impedance signal without the Tyr enzyme printed Au IDE showed fluctuations in the signal versus the increased concentration of Pyrogallol. This biosensor can be used as a promising tool for the analysis of antioxidants.

Recently, Mehmeti and co-workers reported a simple and highly sensitive electrochemical biosensor using laccase immobilized onto a gold nanoparticles/graphene nanoplatelets-modified screen-printed carbon electrode (LACC/AuNP/GNPI/SPCE) to monitor the phenolic antioxidant capacity in beverages (wine and blueberry syrup) [49]. Graphene nanoplatelets (GNPI) were utilized to provide good electronic conductivity and mechanical support for the laccase enzyme, an oxidoreductase capable of oxidizing phenolic compounds. In addition, the current response was maximized due to the synergistic effect caused by the coexistence of gold nanoparticles and graphene nanoplatelets, improving the sensitivity, speed, and stability of the biosensor (Figure 3d). For the preparation of the LACC/AuNP/GNPI/SPCE, graphene nanoplatelets-modified screen-printed carbon electrodes (GNPI/SPCE) were first constructed by printing with carbon ink modified with graphene nanoplatelets on pre-etched ceramic substrates using a semi-automatic printing device. Then, a gold nanoparticle solution (AuNP) was deposited by drop-casting onto the GNPI/SPCE, and enzyme solution were dropped onto the AuNP/GNPI/SPCE for preparation of the laccase-modified electrode. The calibration curve of LACC/AuNP/GNPI/SPCE exhibited a linear range from 4.0 × 10^0^ to 1.3 × 10^2^ μM with a detection limit of 1.5 μM.

#### 2.2.3. Polymers

Polymers have been one of the most promising modifiers in the application of disposable SPEs due to their unique polymeric natures, biocompatibility, stability, and mechanical properties [75]. Polymer-based electrodes have valuable physical-chemical properties as electrode interfaces in electrochemical biosensors. Firstly, due to their high biocompatibility and distinct architecture, polymer-based SPEs can accommodate diverse nanomaterials and biomolecules (e.g., enzymes, receptors) into the polymer matrix, enabling facile alteration of SPE-based biosensors [75]. Secondly, polymers like hydrogel provide a porous matrix that combines catalytically active compounds close to the electrode surface, and these binding properties and porous structure allow pre-concentration of the analyte within the polymer film, which can be significant merit for detection of analytes at trace concentrations [76]. Polymer modification of the electrode surface has been successfully used for reducing the blocking/fouling phenomenon by selective exclusion of interfering species and by protecting the electrode. Sometimes, natural or unknown samples can contain many interferents that influence the result of experiments by adsorbing on the surface or undergoing electrode reactions. Polymer coatings can address these issues [76]. Lastly, conducting polymers, a class of polymer, have recently attracted considerable attention for SPEs due to their particular properties, the most important of which are electrocatalytic effects contributing to increased reaction rate and sensitivity. They have high electrical conductivity due to the delocalized electrons in the polymer chain and good flexibility as printable ink materials (e.g., polyaniline, polypyrrole, polythiophene, etc.) [77,78,79]. Here, several approaches for the determination of antioxidants based on polymeric biosensor are presented.

A study by Chailapakul et al. described a simple and low-cost development of a graphene and polyaniline nanocomposite-modified screen-printed carbon electrode (G–PANI/SPCE) using an in-house screen-printing and inkjet-printing method for the determination of polyphenolic antioxidants in teas [50]. The G–PANI-modified SPCE electrode enhanced the electrochemical sensitivity and had a higher peak current by 2–4 times compared to the bare SPCE. The fabrication of G–PANI/SPCE involved two printing steps. First, to construct the screen-printed carbon electrode (SPCE), a carbon ink was screen-printed onto a polyethylene terephthalate (PET) substrate to make a working electrode, and silver/silver chloride (Ag/AgCl) was printed as a conductive pad. Then, the conductive ink composed of a graphene (G) and polyaniline (PANI) solution was loaded into the cartridge of a piezoelectric inkjet printer and jetted out from the nozzle on the working electrode area of the SPCE. Moreover, ultra-high performance liquid chromatography coupled with the ECD (electrochemical detection) method (UHPLC-ECD) and UHPLC coupled with the UV method (UHPLC-UV) as a standard were compared under the same conditions for the separation and determination of four different antioxidants with this modified electrode. Based on the results, the UHPLC-ECD (G-PANI/SPCE) method exhibited good linearity (linear calibration, 0.01–10 μg/mL) and comparable LODs (limits of detection, 1.4–1.9 ng/mL) for the antioxidants compared to standard and previous reports. This result shows that the G–PANI/SPCE approach is a useful application for the determination of antioxidants in tea samples.

Girault et al. demonstrated an inkjet-printed nano hydrogel coated on the surface of carbon nanotubes (CNT) electrodes to measure antioxidants in red wine and orange juice [18]. They used polyacrylamide/bis (PA) hydrogel as an ink for inkjet printing. They developed an acrylamide/bis ink with high viscosity, good surface tension, and chemical-free for the fabrication of the PA coated CNT electrodes. Briefly, the PA/CNT electrodes were prepared by the Dimatix printer, and four layers of the inks were dropped on a polyimide substrate with fast and efficient polymerization, as shown in Figure 3e–g. First, the nanosilver, then the CNT, and then the insulator, and finally the PA ink were dropped on the polyimide substrate. After the droplet was deposited on the substrate, the photopolymerization of PA was achieved by a UV lamp. The voltammetric analysis of gallic acid (GA), undiluted red wine, ascorbic acid, untreated orange juice, and filtered orange juice samples were analyzed with the PA/CNT electrodes and compared with the bare CNT electrodes and screen-printed carbon paste electrodes (CPEs). Anodic peaks were observed based on the electrochemical oxidation and reduction potential of the samples. For example, gallic acid, one of the antioxidants present in red wine, was analyzed based on the electrochemical oxidation of gallic acid. In conclusion, the PA/CNT electrodes had a more reliable and sensitive electrochemical response compared with the bare CNT electrode even in undiluted samples thanks to the properties of the PA hydrogel thin film coating on the electrode. This electrode showed a new testing approach for the amperometric detection of antioxidants in complex matrices without a matrix effect.

#### 2.2.4. Alternative Screen Printing Techniques

The screen printing technique is a widely used method for fast and easy mass production of disposable and portable electrochemical sensors at a relatively low cost. Nevertheless, the screen printing technique has a few disadvantages, including incompatibility with a non-planar substrate and many microfabrication steps. There are many alternatives to replace the screen printing technique for electrochemical devices, such as xurography, roller-pen writing, stamp contact printing, and laser-scribing. Here, we present a brief overview of these alternative methods. 

Xurography is a prototyping technique of microfluidics that uses a knife plotter for cutting structure in thin foils. Recently, A. Escarpa et al. proposed a low-cost scalable xurography approach for the development of electrochemical microfluidic devices [80]. Customized screen-printed carbon electrodes were integrated into microfluidic channels, and the microfluidic and electrochemical units were fabricated using a digital cutting plotter and screen printing. The analytical capabilities of the devices were demonstrated by the development of an antioxidant capacity assay. Such a xurography combined with a microfluidic device could offer significant progress over current screen printing-based analytical methods (e.g., rapid and low-cost fabrication of Point-of-Care (POC) devices) [81]. However, further details on this tool are not be discussed here because it is considered as another review topic which is deviated from the core of this review.

Roller-pen writing is a method for the deposition of conductive materials on a solid surface. The pen writing method has good advantages compared to other available methods for the preparation of conductive thin films. Firstly, the roller-pen writing method provides a broad choice of the formulation of conducting materials for different substrates than inkjet printing whose formulation of materials is somewhat limited to prevent blocking the printer’s nozzle. This method also makes it possible to directly deposit the conductive inks onto non-planar surfaces, thus overcoming a limitation of conventional screen printing that has to be placed only on flat substrates. Wang et al. designed an enzymatic-ink-based roller pen for direct incorporation of the sensing element into renewable glucose sensor strips [82]. Shrivas et al. also showed a successful application of AgNPs-printed conductive electrodes in cyclic voltammetry measurement using a rollerball pen refilled with AgNPs ink for drawing an electronic circuit on the paper substrate [83]. 

Stamp contact printing is another alternative approach to the fabrication of printed electrochemical sensors. The conductive and insulating inks for diverse electrode patterns can be transferred using various stamps from elastomeric stamps (e.g., PDMS) to rigid substrates. Wang et al. devised versatile stamp transfer electrodes as an alternative to conventional SPEs [84]. They demonstrated that the stamped electrode devices exhibited comparable performance to that of the screen-printed counterparts while addressing their limitations, such as resilience against severe mechanical deformation. The sensors proved usability through the detection of various physiological analytes, including uric acid on the skin.

On the other hand, the laser-scribing technique is utilized as a low-cost, rapid, and facile method to produce laser-scribed electrodes for on-chip electrochemical sensors. This method can directly pattern or synthesize electrode arrays on flexible substrates, which facilitates electrodes’ fabrication process by eliminating many microfabrication steps. Alshareef et al. reported the fabrication of highly efficient laser scribed graphene (LSG) electrodes for flexible electrochemical sensors [85]. The LSG electrodes have characteristics with self-standing porous 3D morphology and abundant edge planes, which enables a higher heterogeneous electron transfer rate of the electrodes than those for similar carbon-based materials. The LSG electrodes exhibited significantly improved electrocatalytic activity toward oxidation of ascorbic acid, dopamine, and uric acid in cyclic voltammetry. The proposed LSG electrodes can offer a great platform for developing printed electrochemical sensors for a wide range of analytical applications.

Overall, an advance in the above-described methods is expected to contribute greatly to the development of low-cost and user-friendly antioxidant screening assays while overcoming the limitations of conventional printed electrode-based electrochemical sensors.

## 3. Printing-Based Antioxidant Therapies

### 3.1. Skin Therapeutics

Antioxidants (e.g., polyphenols, flavanols, oligomeric and polymeric proanthocyanidins, etc.) have many biological activities including anti-inflammatory, anti-carcinogenic, anti-viral, anti-bacterial effects, etc [86,87,88,89,90,91]. Supplementing antioxidants in various therapeutics is a promising approach to defend against the undesirable effects of reactive oxygen species (ROS)-induced oxidative damage in humans. Nowadays, the interest in printing technology for biomedical applications has increased significantly over the past decade [92,93,94]. There are a variety of printing technologies such as stereolithography (SLA), selective laser sintering (SLS), fused deposition modeling (FDM) (or fused filament fabrication (FFF)), digital light process (DLP), etc. Among them, the FDM system is the most common 3D printing method, which utilizes thermoplastic filaments to create a three-dimensional object by heating and then extruding it layer by layer [95]. The most common material used for FDM is poly(lactic acid) (PLA). This is because PVA is a renewable, biodegradable, and biocompatible polymer with high mechanical strength and low coefficient of thermal expansion [96]. In this section, some advances in which antioxidants were applied especially for dermatology using the FDM method are introduced.

**Figure 4:**
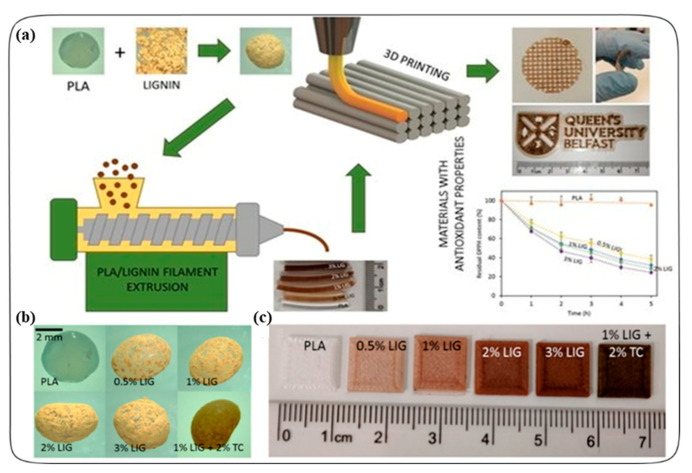
(**a**) Schematic representation of the experimental setup to prepare antioxidant materials used for 3D printed meshes. (**b**) Photographs of poly(lactic) acid (PLA) and PLA-coated pellets. (**c**) Lignin (LIG) and tetracycline (TC) containing squares fabricated using 3D printing. Reprinted from Ref. [39] with permission from MDPI.

Larrañeta et al. prepared a 3D printed antioxidant material for wound healing applications [39]. In this process, lignin (LIG) was used as an antioxidant material, and coated into the poly(lactic acid) (PLA) pellets using castor oil by the hot-melt extrusion method. LIG is a biopolymer and has antioxidant and antimicrobial properties. They also added the antibiotic tetracycline (TC) to the PLA pellets to prevent bacterial infection. The LIG or/and TC-coated PLA pellets were utilized to prepare the filaments needed for making 3D printed materials. This process is shown in Figure 4a. Different shapes (disc and square) of the materials were printed with different weights of the LIG (0.5, 1, 2, and 3% *w/w*) with a fused filament fabrication (FFF) system (Figure 4b,c). In the FFF, the filament was extruded through a heated nozzle and subsequently solidified on a plate. The antioxidant activity of the 3D printed materials was measured using the 2,2-diphenyl-1-picrylhydrazyl (DPPH) radical assay based on the radical scavenging activity of the LIG. The 3D printed materials containing LIG had a higher scavenging activity as the presence of the DPPH concentration decreased over time compared to the control sample (PLA, PLA+Castor oil). The stability studies showed that the materials did not lose weight after 30 days in PBS at 37 °C. The antimicrobial activity of tetracycline loaded PLA/LIG (PLA/LIG (1%)/TC (2%)) 3D printed composite also showed significant bacterial (S. aureus) inhibition compared to the PLA/LIG alone. Furthermore, using this antioxidant PLA/LIG composite material, they prepared a 3D printed mesh for the loading of Curcumin. The meshes were prepared by two-layered arrangements. The first layer contained a PLA/LIG mesh and the second layer was printed using polyvinyl alcohol (PVA). A delayed Cur release was observed due to the PVA film printed in combination with the PLA/LIG mesh. In addition, the PVA film could have a double role, providing a moist environment to the wound while delaying and controlling the CUR release. Overall, the antioxidant and antimicrobial properties of LIG and TC with PLA is advantageous for healthcare application, especially for chronic wound care. Moreover, due to the low price and its versatility of FFF, controllable design of materials or complex geometries can be prepared to meet the specific needs of a patient in hospitals (e.g., customized wound dressings, scaffolds).

Casettari et al. demonstrated the preparation of glycyrrhetinic acid-loaded ethanolic liposomes using a customized 3D-printed microfluidic chip. 18-α-Glycyrrhetinic acid (GA) is a triterpene saponin that exhibits anti-inflammatory and anti-oxidant activities when applied topically to the skin [44]. However, GA has a hydrophobic nature and poor solubility in water, and hence GA-loaded liposomes were adopted to overcome its limited bioavailability. Ethanol was added to the liposome formulations as a skin permeation enhancer. To achieve the optimal liposomal dispersion for topical application, they manufactured a biodegradable and cost-effective PLA 3D-printed microfluidic chip using an FDM 3D printer. The microfluidic chips made it possible to scale up the production of liposomes quickly. This study showed that the optimal liposomal formulation not only presented no cytotoxicity but had better efficiency than that of the conventional formulation containing free GA. The liposomal formulation presented good stability over a storage period of 30 days at 4 °C without significant drug leakage. After hydrogelation of the formulation using xanthan gum, the liposomal hydrogel was evaluated using Franz diffusion cells. The result showed that drug release and permeation were higher in the liposomal hydrogel compared to control formulations. In conclusion, this proposed technique can be a practical and affordable approach for the controlled release of topical dosage forms for hydrophobic bioactive compounds.

### 3.2. Tissue Mimetic 3D Cultures

Three-dimensional (3D) cell culture models have many advantages over conventional two-dimensional cultures. Culturing cells in 3D reproduces the anatomy and physiology of a tissue in physiological conditions, leading to natural cell shapes (ellipsoid/polarized) that are prevalent for cell to cell junctions and a heterogeneous cell interface with the medium [97]. These conditions can influence cellular processes such as differentiation, metabolism, gene expression, and morphogenesis [98,99]. Therefore, creating an advanced 3D culture system becomes an imperative work in a wide range of fields from drug discovery to tissue engineering. A variety of materials have been used for the preparation of 3D cell cultures. Antioxidants is one of the attractive materials for 3D cultures due to their free-radical scavenging activities and supplementary benefits like their anti-inflammatory effect, etc. Recently, a novel 3D scaffold development was proposed to detoxify H_2_O_2_ oxidative stress for robust cell growth and angiogenesis. Cho et al. developed a catalase-entrapped large-sized 3D scaffold which can reduce hydrogen peroxide (H_2_O_2_)-induced damage in tissue regeneration [40]. The accumulation of excess H_2_O_2_ released by cells is one of the primary issues that should be solved for successful large tissue-engineered grafting. Catalase enzymes in the gel (a mixture of alginate and decellularized adipose tissue matrix (DAT) hydrogel) were coated onto a 3D printed polycaprolactone (PCL) scaffold 10 × 10 × 6 mm^3^ with a rectangular pore size of 600 μm and a line width of 200 μm. The catalase detoxified the H_2_O_2_, thereby generating oxygen and water as byproducts, which prevented the local microenvironment from converting into a hypoxic region and hence promoted cells to survive. To evaluate gel stability coated in 3D PCL scaffolds, the gel-mass detachment assay was performed for 14 days, and the 3D scaffolds coated with DAT-alginate gel provided a better coat to the PCL scaffold with its ability to release catalase in a sustained manner. In vitro results showed that catalase released from the 3D scaffold rescued human turbinate mesenchymal stem cells (hTMSCs) from H_2_O_2_ induced oxidative stress. An in vivo study of subcutaneous-implanted scaffolds in rats demonstrated both reduced inflammation (≥40%) and increased tissue growth (≥45%) and the induction of angiogenesis (≥40%). This model is a promising candidate for the regeneration of damaged tissues. 

Furthermore, Osterberg et al. devised a cellulose nanofibril-alginate-spherical colloidal lignin particle (CNF-alginate-CLP) nanocomposite scaffold fabricated by 3D printing for a three-dimensional printed cell culture model [42]. Cellulose nanofibril (CNF) hydrogels have gained attention as a good material suitable for 3D printing cell cultures and tissue engineering due to its hydration capacity, biocompatible and shear-thinning properties. Combination cellulose nanofibril hydrogels with alginate enabled a printed scaffold cross-linked in the presence of calcium. Furthermore, the addition of spherical lignin nanoparticles not only provided antioxidant properties to the CNF-alginate scaffold but also improved the printing resolution and shape stability due to its viscosity. For the production of cylindrical scaffolds with a diameter of 1.5 cm and a height of 2 cm, biomaterial inks including CNF hydrogel, alginate, and CLP dispersion were printed on polypropylene Petri dishes by the BIOX 3D bioprinter. The CNF-alginate-CLP scaffolds represented shape stability after storage in cell culture medium up to 7 days with demonstrating a high water-retaining capability. In the cell viability test, the hepatocellular carcinoma cell line (HepG2) grown in the CNF-alginate-CLP based printed scaffold showed consistent proliferation, suggesting that this CLP-containing scaffold have great potential as a good candidate for soft-tissue engineering applications in regenerative medicine.

### 3.3. Bone Tissue Engineering

3D-printed bone tissue engineering is an attractive approach in alternative treatments for bone cancer or orthopedic trauma. 3D-printed bone scaffolds can replenish post-surgical critical-sized defects of cancer patients [100,101]. Additionally, patient-specific anatomical implants can be fabricated by 3D printing technology. The 3D printing technology is very suitable for manufacturing bone tissue scaffolds due to its many strengths including high speed, precision, and directly controllable fabrication by computer-aided design (CAD). Conventionally, calcium phosphate ceramic scaffolds are mainly used as an inorganic component of bone tissue, especially hydroxyapatite (HA) and β tricalcium phosphate (β-TCP) because of its osteoinductivity, osteoconductivity, and biodegradability [38,102,103]. Porosity inside scaffolds is also an important factor because it can influence not only the ingrowth of new bone but vascularization by providing a space for cell growth and a nutrient supply [104]. Although a variety of studies on 3D-printed bone tissue engineering have been presented over the last decade, antioxidants recently begun to be used in some reports for the development of 3D-printed bone tissue scaffolds. 

**Figure 5:**
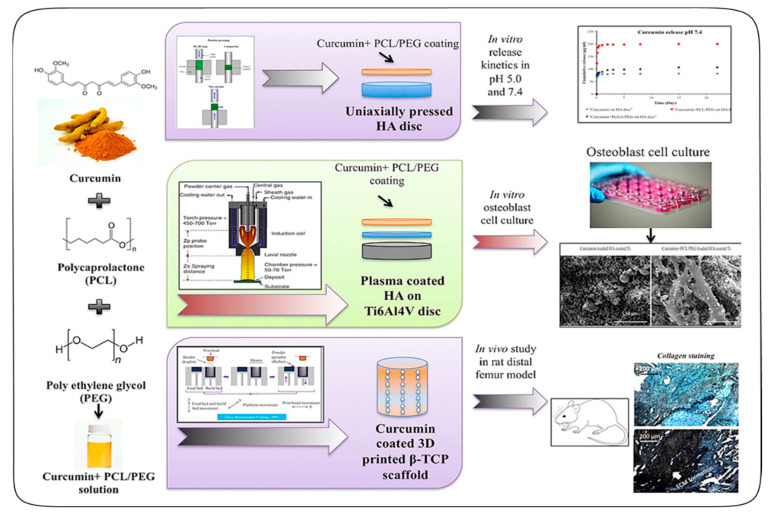
Schematic illustration of the curcumin-polycaprolactone (PCL)-poly(ethylene glycol) (PEG) polymeric system and curcumin-coated 3D printed tricalcium phosphate (TCP) scaffold for in vitro and in vivo bone generation. Reprinted with permission from Ref. [38]. Copyright (2018) Elsevier.

Bose et al. proposed for the first time a 3D printed interconnected macroporous β-TCP scaffolds, which were loaded with curcumin-PCL-PEG for bone regeneration, as shown in Figure 5 [38]. Curcumin is a phenolic compound, which has antioxidant, anti-inflammatory, and anticancer activities, but its use has been limited for in vivo chemotherapy due to its hydrophobicity. By encapsulating curcumin into a poly (ε-caprolactone) (PCL)—polyethylene glycol (PEG) polymeric system, the bioavailability of curcumin could increase. The curcumin-PCL-PEG system also enabled the controlled release of curcumin. In a cell viability assay, the results showed that the curcumin-PCL-PEG polymeric system assisted osteoblasts to proliferate in HA plasma coated Ti6A14v samples. Subsequently, for an in vivo study, cylindrical scaffolds 3.2 mm in diameter and 5 mm in height along with 400 μm sized pores were produced by the ProMetal 3D printer, and the curcumin-polymer solution was then dropped onto the entire scaffolds to achieve the desired drug loading. The results showed that the curcumin coated bone-like scaffolds enhanced in vivo bone formation from 29.6% to 44.9% compared to the control TCP scaffold in rats. This curcumin-loaded scaffold showed a superb early wound healing and osteogenesis capability. Thus, this work established a novel bifunctional bone tissue engineering scaffold, which not only eradicated bone cancer cells but also promoted bone formation within the porous scaffold, offering a promising drug delivery strategy to treat bone defects after tumor resection.

Similarly, Bose et al. also reported the fabrication of 3D printed-bone tissue cylindrical scaffolds with an interconnected porosity loaded with liposome-encapsulated curcumin [41]. By incorporating curcumin into liposomes, the bioavailability of curcumin could increase. The 3D printed-bone tissue scaffolds were built from tricalcium phosphate (TCP) ceramic which is a good stimulator of bone formation and repair. The scaffold 3.2 mm in diameter and 5 mm in height along with square-shaped pores and 400 μm in size were fabricated using a binder jet printer. The MTT cell viability results showed that the curcumin-encapsulated liposome-loaded 3D printed scaffolds enhanced the viability of healthy bone (osteoblast) cells while inhibiting the proliferation of bone cancer (osteosarcoma) cells compared to the control 3D printed TCP scaffold. The scaffold implantation at the femoral defect of rats resulted in enhanced bone formation after 6 weeks. This liposomal curcumin-loaded 3D printed porous scaffold has its potential as one of the novel bone graft substitutes for bone regeneration after surgical resection of a tumor.

## 4. Conclusions

Nowadays, there is a growing interest in substances exhibiting an antioxidant activity, which are supplemented to humans as food components or as specific preventive nutraceuticals. Consequently, antioxidants have become an integral part of healthcare industries and at the same time food preservation technology. Over the past few decades, the emergence of diverse printing technologies has proved revolutionary in screening antioxidants. Screen printing technology has opened new prospects in producing straightforward and cost-effective sensing devices. Screen printing technology makes it possible to mass-produce inexpensive, reproducible, and sensitive disposable electrodes, and SPEs have been applied in miniaturized and portable devices for specific purposes, which fits with the current trend of tests done in situ, in real-time, or inline. The enormous versatility demonstrated by SPEs is based on the wide range of modifications done on the electrodes, as presented in the literature. The incorporation of particular materials into SPEs enables higher sensitivity, selectivity, and stability. Despite these attractive advantages of SPE-based platforms, screen printing technology has a limitation that is incompatible with non-planar substrates, which hinders further research progress on flexible printed electronics. In this regard, the development of alternative screen printing technologies (i.e., roller pen writing and stamp contact printing) can overcome the limitation by providing high flexibility and resilience against mechanical deformation during the printing process. On the other hand, inkjet-printed free-radical scavenging assays are another promising printing-based antioxidant screening tool. This methodology is a cost-effective approach due to its small consumption of reagents, which is especially advantageous for the screening of high-priced active compounds. Additionally, the inkjet printing system meets the increasing demands for labor-free, high-speed, and economical analyses breaking away from traditional well-plate methods. Despite these conveniences, the inkjet printing-based assay has a shortcoming. Ink formulation with easy flow to prevent clogging of inkjet nozzle often make it difficult to analyze antioxidants in viscous samples or deposit inks on non-planar substrates. 

Lastly, we discussed the latest findings on 3D printing-based antioxidant therapies in the field of biomedical engineering. Among the many 3D printing techniques, the FDM method, the most common one, has been used to create diverse objects (e.g., porous scaffolds, hydrogels, meshes, and fibers) suitable to improve the healthcare of traumatic and diseased patients. To promote the development of tissue engineering and regenerative medicine, it is essential to better understand and provide a favorable microenvironment around tissues and organs. The improvement of the microenvironment could be achieved by applying beneficial ingredients such as antioxidants to biomaterials that are used to fabricate scaffolds. Although the investigation into the use of antioxidants in tissue engineering models has only begun in recent years, antioxidant-combined therapy is highly desirable for the organization of cells and the management of oxidative stress, which is beneficial in augmenting and repairing tissue function in the body. This 3D printing-based antioxidant therapy has the potential for multiple applications. For instance, catalase-entrapped scaffolds would be very helpful to replace the ischemic tissue of patients by detoxifying H_2_O_2_ and generating oxygen in the microenvironment. In addition, the combination of 3D printing’s versatility with a promising drug delivery strategy can offer advanced properties in tissue engineering or regenerative medicine, such as a prolonged antioxidant activity and a tailorable design for patients on demand. For example, a recent finding such as the successful fabrication of liposome-encapsulated curcumin-loaded calcium phosphate scaffolds can be a good candidate for bone detects. In conclusion, the printing technology-based approach will be an excellent platform to open the door to the next generation of antioxidants screening tools and therapies.
